# COVID-19 associated with AIDS-related disseminated histoplasmosis: a case report

**DOI:** 10.1177/0956462420957518

**Published:** 2020-09-09

**Authors:** Mauro Bertolini, María Felicitas Mutti, José AE Barletta, Adriana Falak, Daniel Cuatz, Alicia Sisto, Martín A Ragusa, Nigel Osvaldo Fernandez Claros, María José Rolón

**Affiliations:** 1 Infectious Diseases Division, Hospital Juan A. Fernández, Buenos Aires, Argentina; 2 Internal Medicine Division, Hospital Juan A. Fernández, Buenos Aires, Argentina

**Keywords:** HIV, AIDS, COVID-19, histoplasmosis, opportunistic infections

## Abstract

Limited information is available concerning the coexistence of COVID-19 and opportunistic infections in people living with HIV. The possible association of COVID-19 with AIDS-related respiratory diseases should be considered, particularly in patients with advance immunosuppression. We report the case of a male patient with AIDS-related disseminated histoplasmosis associated with COVID-19.

## Introduction

While universal antiretroviral therapy (ART) is recommended by the World Health Organization (WHO)^1^ to all people living with HIV (PLHIV), regardless of their immune status, only 60% of PLHIV receive sustained ART worldwide^2^; and opportunistic infections (OIs) continue to cause significant morbidity and mortality, particularly in low-and middle-income countries.^1^

Disseminated histoplasmosis (DH) is caused by the dimorphic fungus *Histoplasma capsulatum*, endemic to the central and south-central United States and to Latin America. In these areas, the annual incidence rate may approach 5% among PLHIV,^3^ affecting particularly individuals with CD4 count <150 cells/mm^3^.^1^

Coronavirus disease 2019 (COVID-19) is a respiratory infectious disease caused by the severe acute respiratory syndrome coronavirus 2 (SARS-CoV-2).^4^ On March 11, 2020, COVID-19 was defined as a pandemic by the WHO.^5^ It is not entirely clear how PLHIV are affected by COVID-19, particularly in the setting of profound AIDS-related immunosuppression and coexistence with HIV-related OIs. We report the case of a male patient presenting with AIDS-related DH associated with COVID-19.

## Case report

**Figure 1.**
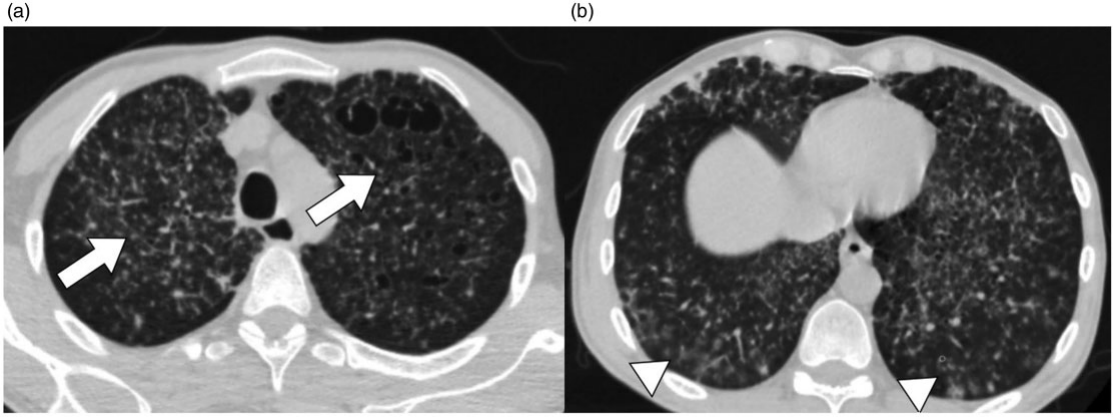
Chest CT-scan (a) Emphysema (arrow) associated with miliary-pattern infiltrates (arrowhead) (b) Bilateral peripheral multifocal ground-glass opacities (arrowheads).

A 43-year-old man with a history of HIV infection and poor adherence to ART presented with cough and dyspnea of acute onset, associated with fever, night sweats, abdominal pain and diarrhea of 1-month duration. Physical examination revealed hypoxemia (pulse oximetry on room air was 93%) and generalized ulcerated skin lesions, as well as bilateral cervical, supraclavicular and inguinal tender adenopathies, oral thrush and hepatomegaly. Generalized hypoventilation and diffuse crackles were found on respiratory examination. Laboratory tests showed ferritin 1500 ng/mL (normal range: 23.9–336.2 ng/mL), D-dimer 431 ng/mL (normal range: <230 ng/mL), C-reactive protein 16 mg/dL (normal range <1.0 mg/dL), LDH 380 U/L (normal range 120–246 U/L), mild transaminitis and a white blood cell count within normal range except for mild lymphopenia. CD4 cell count was 16.3 cells/mm^3^ (3.7%). Chest CT scan demonstrated miliary-pattern infiltrates and bilateral peripheral multifocal ground-glass opacities (Figure 1).

Skin lesion scarification was carried out, with Giemsa staining evidencing the presence of *Histoplasma capsulatum*-like intracellular yeasts. Due to local circulation of COVID-19, SARS-CoV-2 RT-PCR was performed on a nasopharyngeal swab sample collected from the patient and he was admitted to the hospital with a diagnosis of DH. Since liposomal amphotericin was not available, treatment with intravenous amphotericin B deoxycholate at 1 mg/kg/day was administered. The patient remained hemodynamically stable and his oxygen requirements decreased during the course of follow-up. Forty-eight hours after the patient’s admission, his skin culture and lysis-centrifugation blood culture grew *H. capsulatum* and a detectable SARS-CoV-2 RT-PCR result was obtained from the sample taken at hospital admission (amplifying sequences from N-gene and E-gene, with a cycle threshold [Ct] of 36).^6^ Although lopinavir/ritonavir and hydroxychloroquine were considered as possible off-label treatments for COVID-19 at the time,^7,8^ due to concerns about potential toxicity and drug-drug interactions with antifungal therapy, and since the patient’s clinical status had improved significantly, we decided to withhold the specific treatment of SARS-CoV-2.

The clinical course was favorable. After one week of hospitalization and being afebrile for 48 hours, the patient had a new nasopharyngeal swab sample taken and this time the SARS-Cov-2 virus was not detected with RT-PCR. The antifungal therapy was switched to itraconazole (200 mg PO TID for 3 days, then 200 mg PO BID) after 14 days of intravenous treatment and ART was re-started with tenofovir disoproxil fumarate/emtricitabine plus dolutegravir. The patient was subsequently discharged and then, lost to follow up.

## Discussion

Even though limited data are available regarding the clinical course and prognosis of COVID-19 in PLHIV, this population does not seem to be disproportionately affected by it, in terms of incidence or frequency of severe disease.^9,10^ Nevertheless, recent studies have shown higher risk of death from COVID-19 in PLHIV, irrespective of viral suppression.^11^ It should be noted that in the case of PLHIV with advanced disease and profound immunosuppression, COVID-19 may coexist with other AIDS-related conditions, including respiratory OI,^4^ which could lead to difficulties in diagnosis, clinical course, patient management and increased morbidity and mortality.

It has been hypothesized that immunosuppressed patients may have prolonged SARS-CoV-2 viral shedding.^12,13^ However, our patient showed what appears to be a rapid viral clearance in his upper respiratory tract, even in the absence of specific treatment; but since the symptoms of histoplasmosis could have overlapped with those related to COVID-19, the exact date of COVID-19-related symptoms onset could not be precisely defined; therefore, DH may have appeared during the resolution of the previous COVID-19 infection.

## Conclusion

To the best of our knowledge, this is the first case of COVID-19 presenting with AIDS-related DH, reported in the medical literature so far. The possible association of COVID-19 with respiratory OI should be strongly considered in patients with advanced immunosuppression, particularly in areas significantly affected by the pandemic. Further research regarding epidemiology, clinical features, prognosis of COVID-19 in PLHIV and possible association with AIDS-related OI is warranted.
